# Supplementary figures and images for: Cell-cell adhesion regulates Merlin/NF2 interaction with the PAF complex
Source: PLoS One. 2021 Aug 23;16(8):e0254697. doi: 10.1371/journal.pone.0254697 (PMC8382200; doi:10.1371/journal.pone.0254697)

## Slide 1
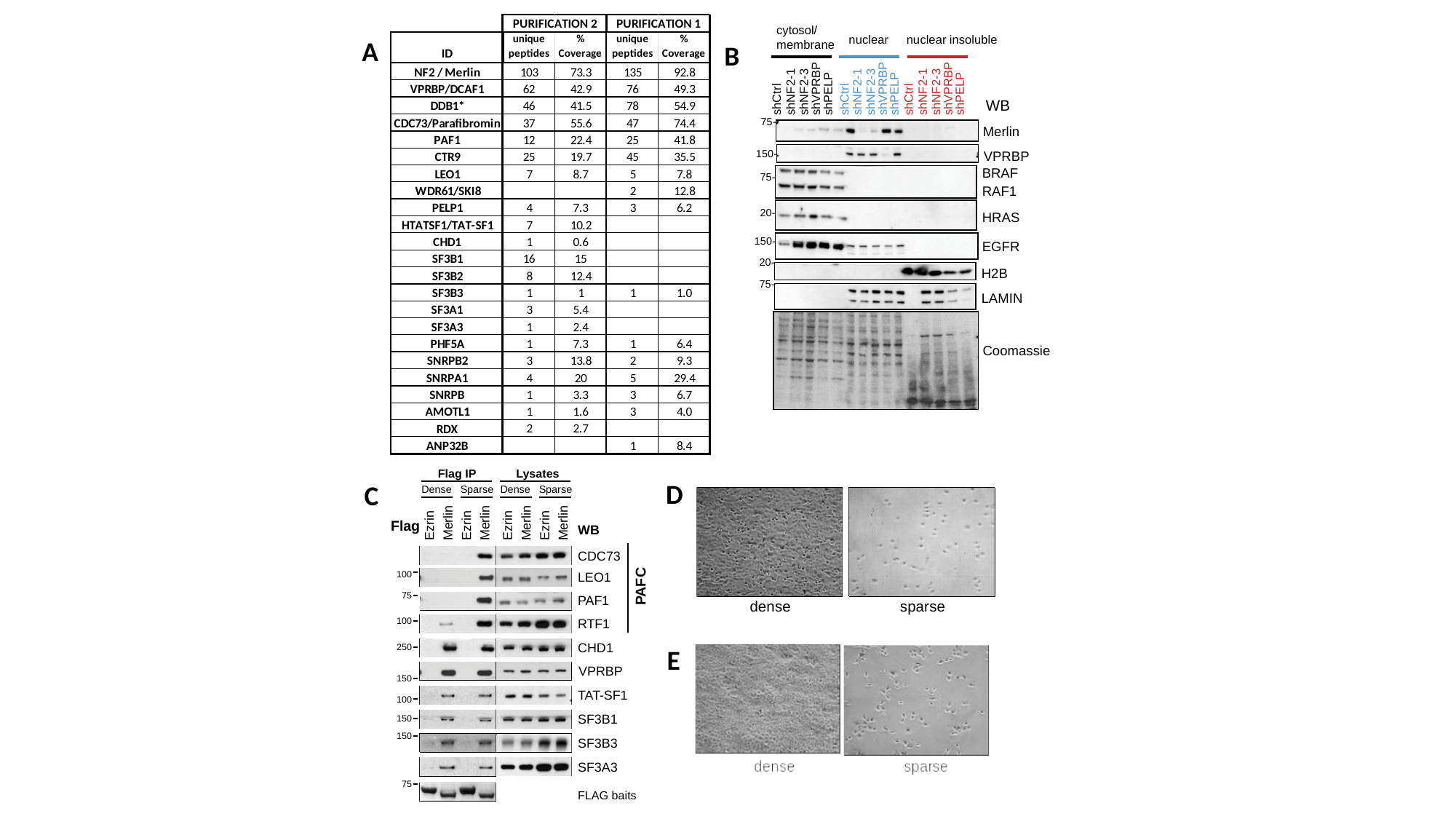

A
B
D
C
E

Supplement: S1 Fig — (A) Proteins identified by mass spectrometry after 2 independent Merlin affinity purifications from HEK293T. * Some peptides for no DDB1 were also found in the SHOC2 control bait used. (B) Merlin resides primarily in the nuclear fraction. IOMM-Lee cells expressing various shRNAs were fractionated into a cytoplasmic/membrane, nuclear and nuclear insoluble fractions and proteins detected by Western blot. The shRNA knockdown of Merlin and Vprbp serve as specificity control for the immunoblot. BRAF/CRAF are used as examples of cytosolic proteins whereas HRAS and EGFR are examples of membrane proteins. Some EGFR can also be detected in the nucleus consistent with previous observations (73). Histone H2B is a marker for nuclear insoluble fraction. (C) Merlin’s interaction with the PAFC is inhibited in confluent cells. Flag IPs from dense or sparse IOMM-Lee cells stably expressing Flag-Ezrin or Merlin were probed with the indicated antibodies. (D) Representative images of cell densities of IOMM-Lee cells at time of experiment in C. (E) Representative images of cell densitis in HMLE cells in experiment in Fig 1G. (PPTX) [file pone.0254697.s001.pptx]

## Slide 1
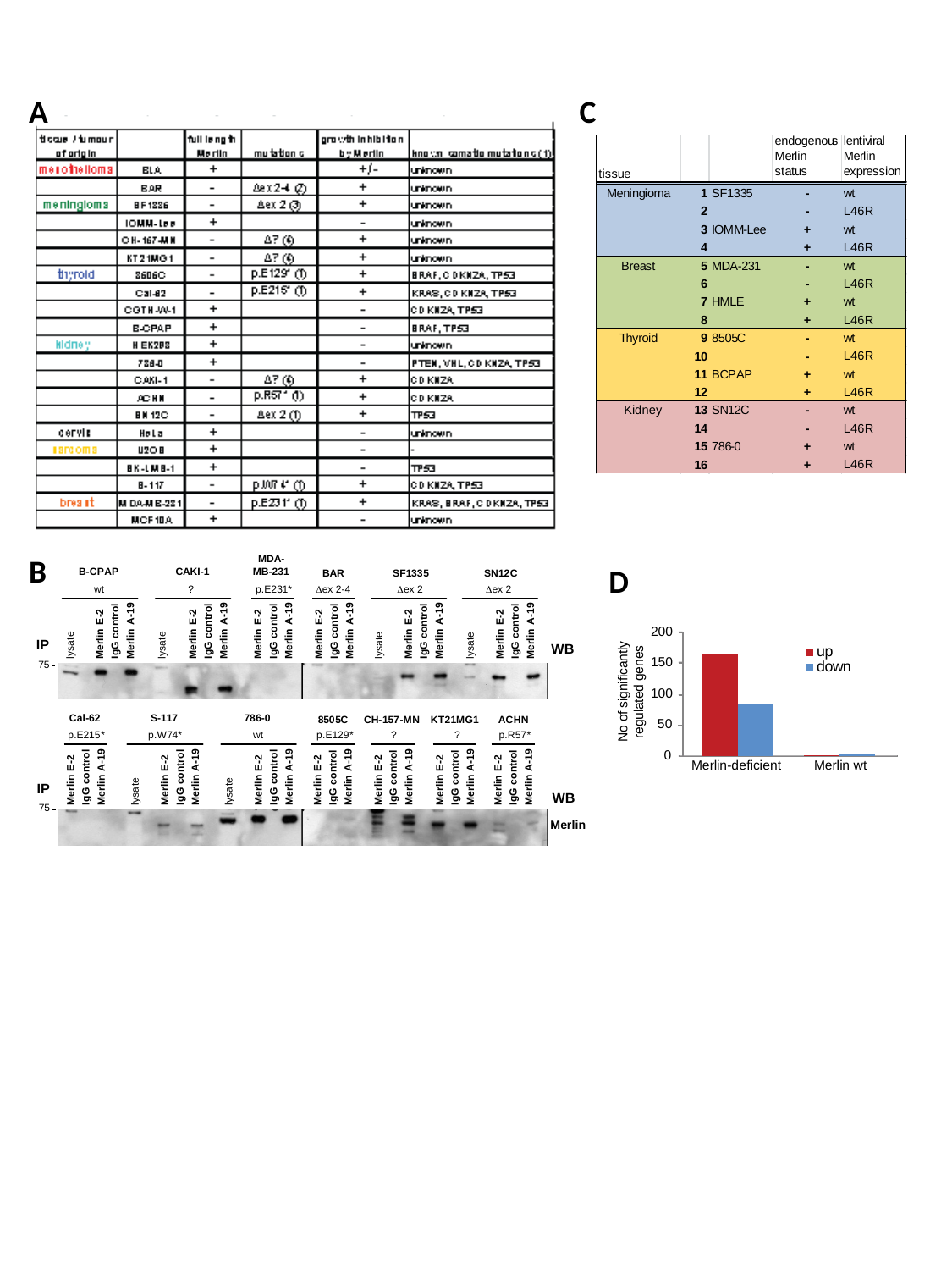

A
C
B
D

Supplement: S2 Fig — (A) Summary of proliferation studies for all tested cell lines in Fig 5. Known NF2 mutations as well as other somatic mutations are listed. (1) COSMIC database, (2) (74), (3) M. Giovannini personal communication, (4) see S2B. (B) Characterization of Merlin expression by IP-wb. Merlin was IPed using antibodies directed against the N-terminus (A-19) and the C-terminus (E-2) and detected by wb using an anti-C-terminus antibody (Bethyl). Cal62, 8505C and ACHN show no Merlin protein expression, whereas CAKI-1, SF1335, SN12C, CH-157-MN, KT21MG1 express shorter protein products consistent with splice site mutations. (C) Experimental design of gene expression microarray experiment in tumour cell lines. Merlin deficient and Merlin wild type cell lines from 4 different tissue types were infected with lentiviruses expressing either wild type or L46R Merlin, RNA was isolated 48 hrs later and analysed using GeneChip® Human Gene 2.0 ST Arrays (Affymetrix). (D) Merlin expression regulates gene expression in Merlin deficient but not Merlin wild type cells. Number of significantly regulated genes (q≤ 0.05, fold change >1.5) identified by microarray. (PPTX) [file pone.0254697.s002.pptx]

## Slide 1
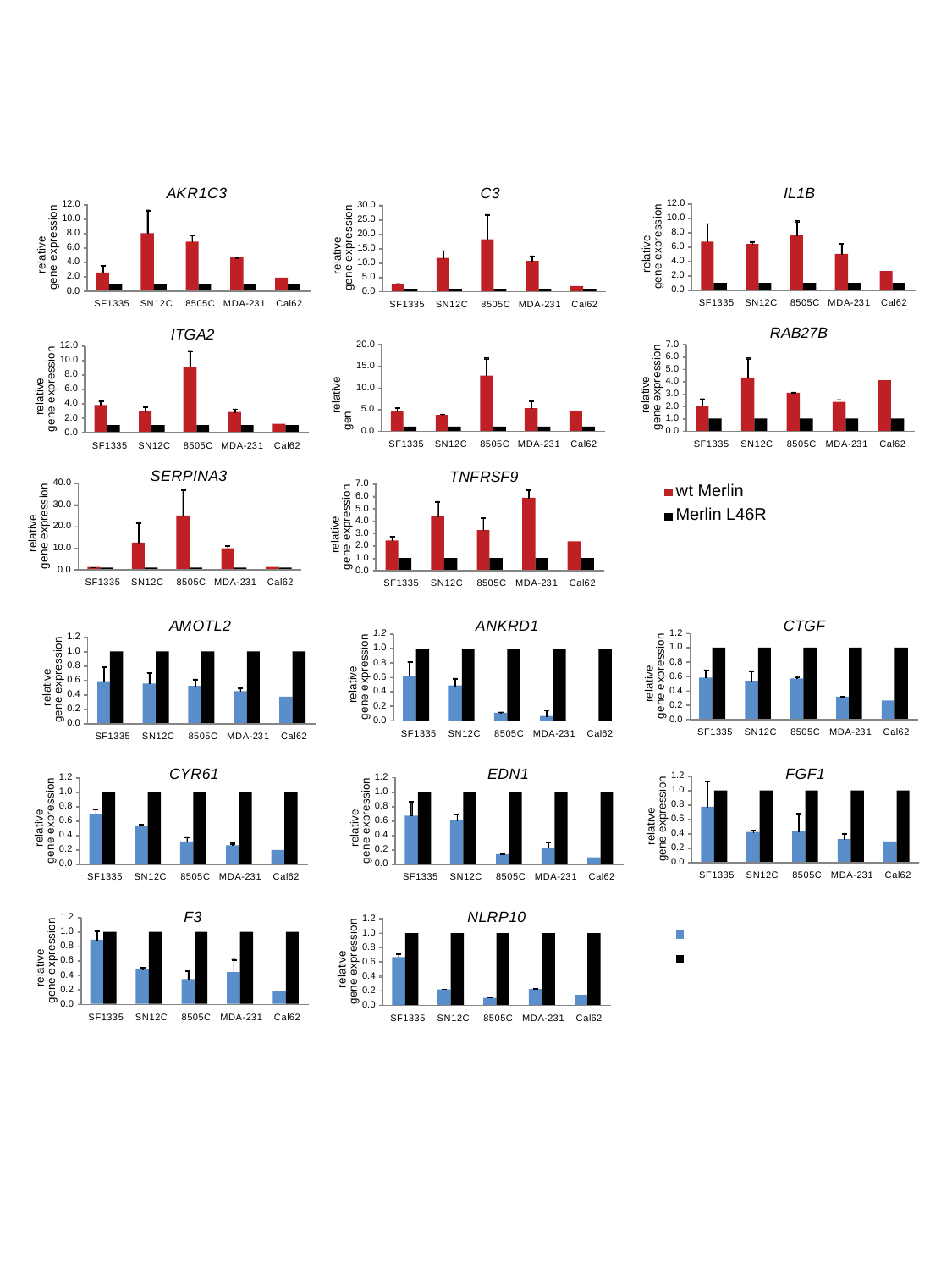

Supplement: S3 Fig — cDNA from cells as in S2C was analysed by qPCR. Data is represented as gene expression relative to actin (ACTB) and normalized relative to Merlin L46R mutant. Error bars indicate SD from three independent experiments. Cal62 were not used in the array experiment, however data from one experiment is shown. Genes upregulated by Merlin expression are shown in red, downregulated in blue. (PPTX) [file pone.0254697.s003.pptx]

## Slide 1
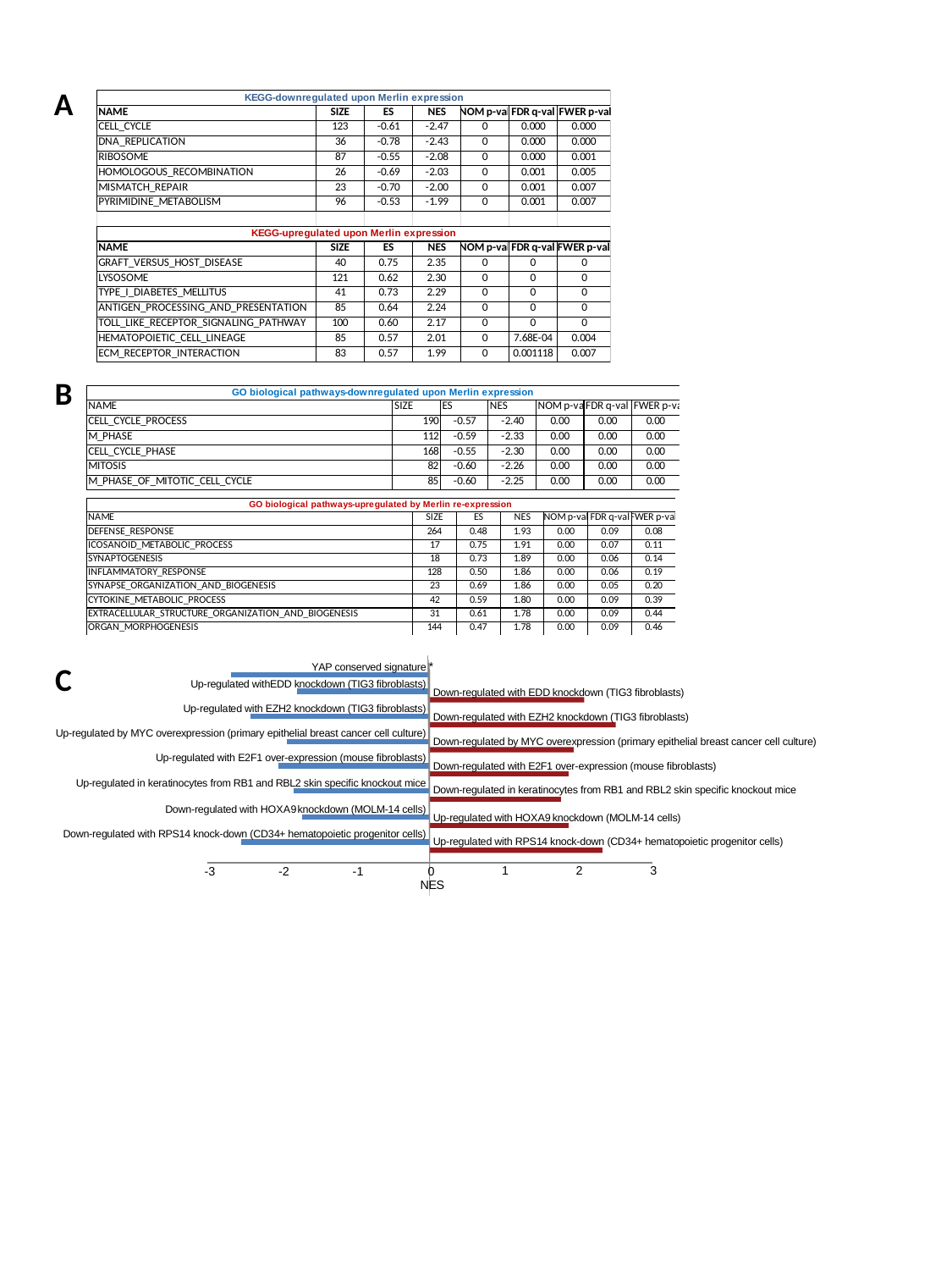

A
B
C

Supplement: S4 Fig — (A, B) Gene Set Enrichment Analysis (GSEA) of Merlin re-expression microarray results compared by KEGG (A) or GO biological pathways (B). Top 5–8 up- and down-regulated gene set pathways shown. (C) Selected transcriptional signatures from Oncogenic Signatures database associated with Merlin hypersensitivity by GSEA. * note that YAP conserved signature only contains genes upregulated by YAP expression. NES, normalized enrichment score. (PPTX) [file pone.0254697.s004.pptx]

## Slide 1
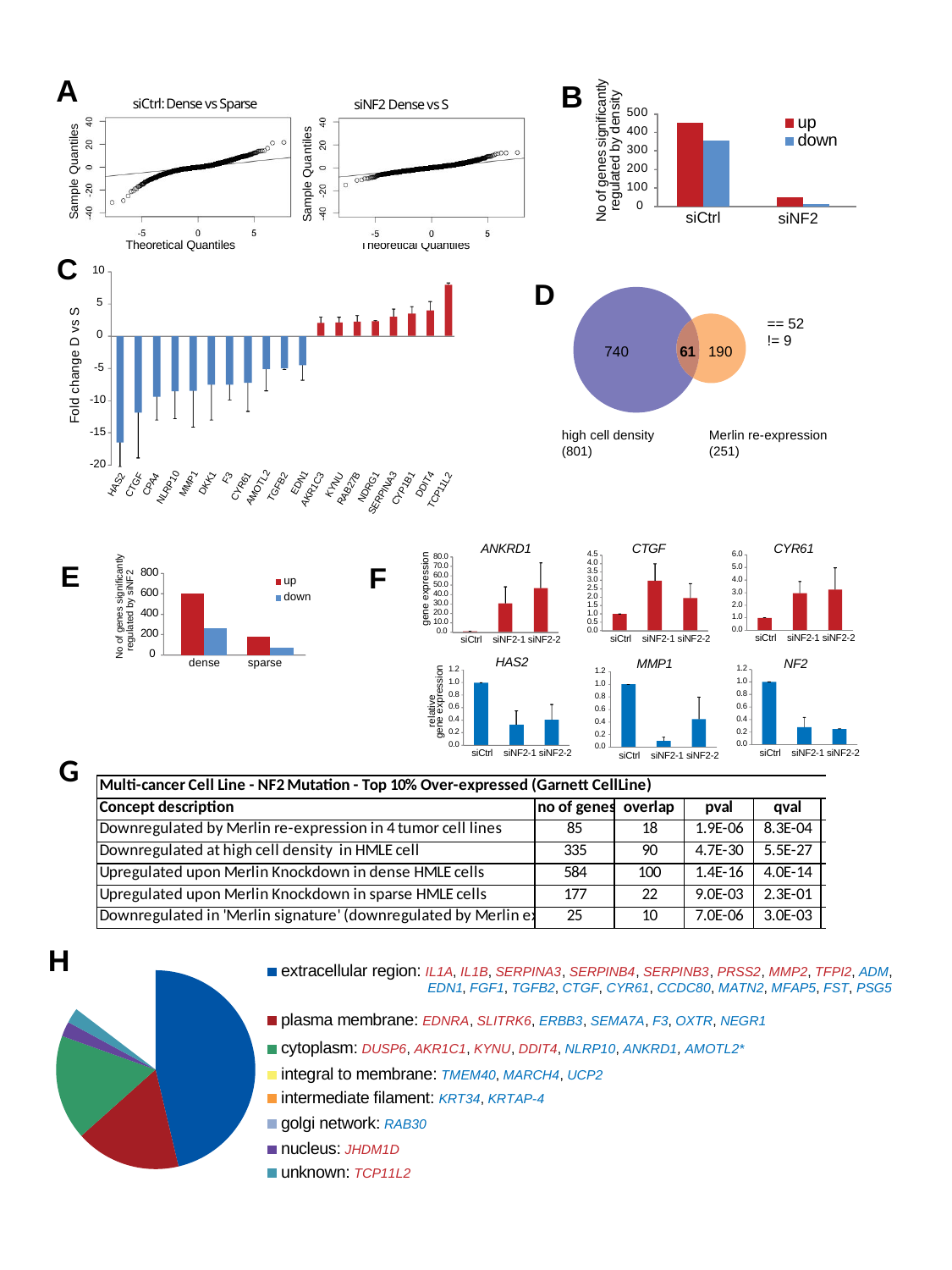

G

Supplement: S5 Fig — (A) Gene expression changes in dense (D) and sparse (S) HMLE cells 3 days after transfection with scramble (Ctrl) or NF2 siRNAs were analyzed by microarray. siControl cells show greater dispersion of limma t-values compared to the theoretical null distribution (black line). (B) As in A but showing number of significantly regulated genes (p≤ 0.05, fold-change >1.5). (C) Validation by RT-qPCR of representative genes regulated by cell density in HMLE cells. Fold change in dense compared to sparse cells. Error bars indicate SD from n = 3. (D) Overlap between the Merlin re-expression and high cell density signatures. The majority of genes (85%, 52 of 61) are regulated in the same direction (= =), while 9 are regulated in the opposite manner (! =). (E) Merlin knockdown has a greater effect on gene expression in dense cells. Number of significantly regulated genes (q≤ 0.05, fold change >1.5) is shown. (F) Validation by RT-qPCR of representative genes regulated by Merlin knockdown in dense HMLE cells with two independent siRNAs. Gene expression relative to GAPDH and fold change normalized to siCtrl. Error bars indicate SD from n = 3. (G) Merlin and high cell density signatures detect NF2 mutational status in the Garnett et al multi-cancer cell line study using the Oncomine database. Significance and overlap shown. (H) Merlin core gene signature is enriched in extracellular and plasma membrane proteins. Merlin core signature is defined as genes regulated in the opposite direction upon Merlin re-expression in 4 tumour cell lines and knockdown in dense HMLE cells. Red indicates positively regulated by Merlin (downregulated by knockdown and upregulated by re-expression), blue negatively regulated by Merlin (upregulated by knockdown and downregulated by re-expression). Protein location based on Gene ontology. (PPTX) [file pone.0254697.s005.pptx]
