# Supplementary material for: Cell-cell adhesion regulates Merlin/NF2 interaction with the PAF complex
Source: PLoS One. 2021 Aug 23;16(8):e0254697. doi: 10.1371/journal.pone.0254697 (PMC8382200; doi:10.1371/journal.pone.0254697)
Supplement: S6 Fig — (A). Gene Ontology term enrichment of proteins co-purifying with PAFC subunits. TAP6-CDC73, PAF1 and LEO1 were expressed and affinity purified from either HT1080 (Merlin wild type) or MDA-MB-231 (Merlin deficient) cells. Co-purifying proteins identified by mass spectrometry were subjected to functional annotation analysis with GO biological process, cellular component and molecular function terms using DAVID (72). Terms correspond to GO:000368, GO:0098609, GO:0016593, GO:0006397 and GO:0000398. (B). Venn diagram showing overlap of proteins identified in HT1080 and MDA-MB-231. See S11 Table for full lists. (C). Venn diagram of proteins identified by AP-MS with each bait (CDC73, LEO1 and PAF1) in either HT1080 or MDA-MB-231 cells. (PPTX) [file pone.0254697.s006.pptx]

## Slide 1
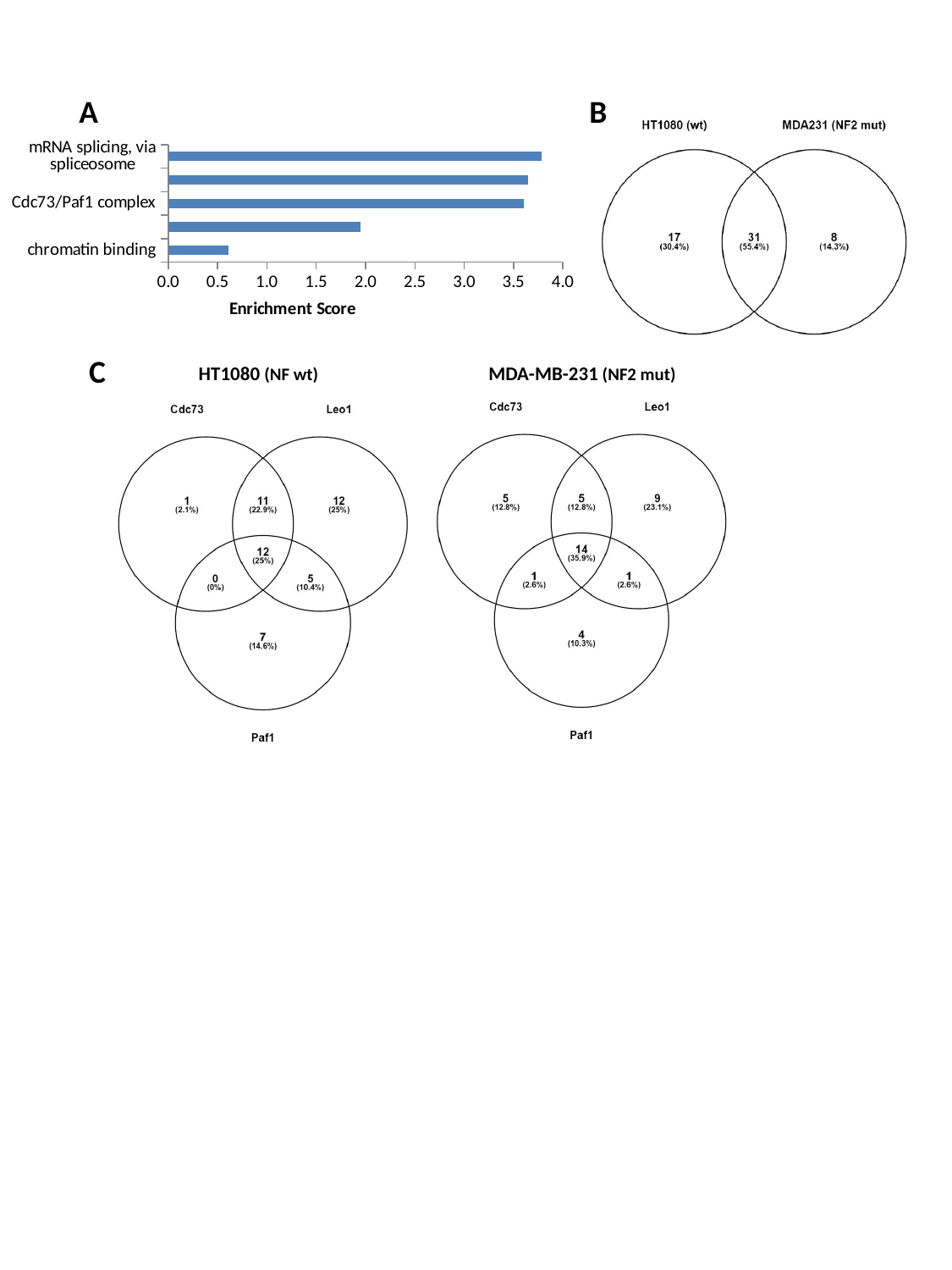

A
B
### Chart
| Category | Enrichment Score: Medium |
|---|---|
| chromatin binding | 0.61281666922706 |
| cell-cell adhesion | 1.95191464630697 |
| Cdc73/Paf1 complex | 3.60838582673983 |
| mRNA processing | 3.6460239826443 |
| mRNA splicing, via spliceosome | 3.78610308658061 |C
HT1080 (NF wt)
MDA-MB-231 (NF2 mut)
