# Supplementary material for: Cell-cell adhesion regulates Merlin/NF2 interaction with the PAF complex
Source: PLoS One. 2021 Aug 23;16(8):e0254697. doi: 10.1371/journal.pone.0254697 (PMC8382200; doi:10.1371/journal.pone.0254697)
Supplement: S7 Fig — At the membrane, Merlin associates with cell-cell contact associated proteins such as a-catenin at adherens junctions and AMOT proteins at tight junctions whereas FAT cadherins may associate with the CDC73 subunit of the PAF complex at distinct adherens junctions. Merlin at the membrane primarily functions to regulate LATS-dependent phosphorylation and inactivation of YAP/TAZ. Upon Merlin inactivation (e.g. upon loss of cell-cell contacts), YAP/TAZ translocates to the nucleus to mediate TEAD dependent recruitment of Pol II and transcription initiation. In the nucleus, Merlin interacts with the CRL4VPRBP E3 ubiquitin ligase (10) and with proteins involved in transcription elongation and RNA processing including the PAFC, CHD1, RTF1 and TAT-SF1 (this study). Through these interactions, Merlin regulates post-initiation events such as pause release, elongation rate and/or RNA processing in at least a subset of target genes. Merlin could use CRL4VPRBP mediated ubiquitination of some associated proteins (such as CDC73 and/or other CRL4VPRBP substrates) to regulate their properties and/or function within this macromolecular complex. Nuclear localization of FAT1 cytoplasmic tail and association with transcription regulators has been reported [59, 63] and the CDC73 interaction with FAT could also take place in the nucleus. AMOT also has dual membrane/cytoplasm and nuclear roles and is required for YAP transcriptional activity of some target genes [75, 76]. ZO-2, another tight-junction associated protein also interacts with YAP in the nucleus [77]. It is therefore possible that separate membrane/cytoplasm and nuclear functions of several of its components is a common feature of the Hippo pathway that allows for independent layers of regulatory control. Note that reported additional interactions of FAT with Hippo pathway components are not shown [76, 78] but also suggest multiple layers of regulation of the Hippo pathway by FAT proteins. Merlin mays also cooperate/antagonize w [file pone.0254697.s007.pptx]

## Slide 1
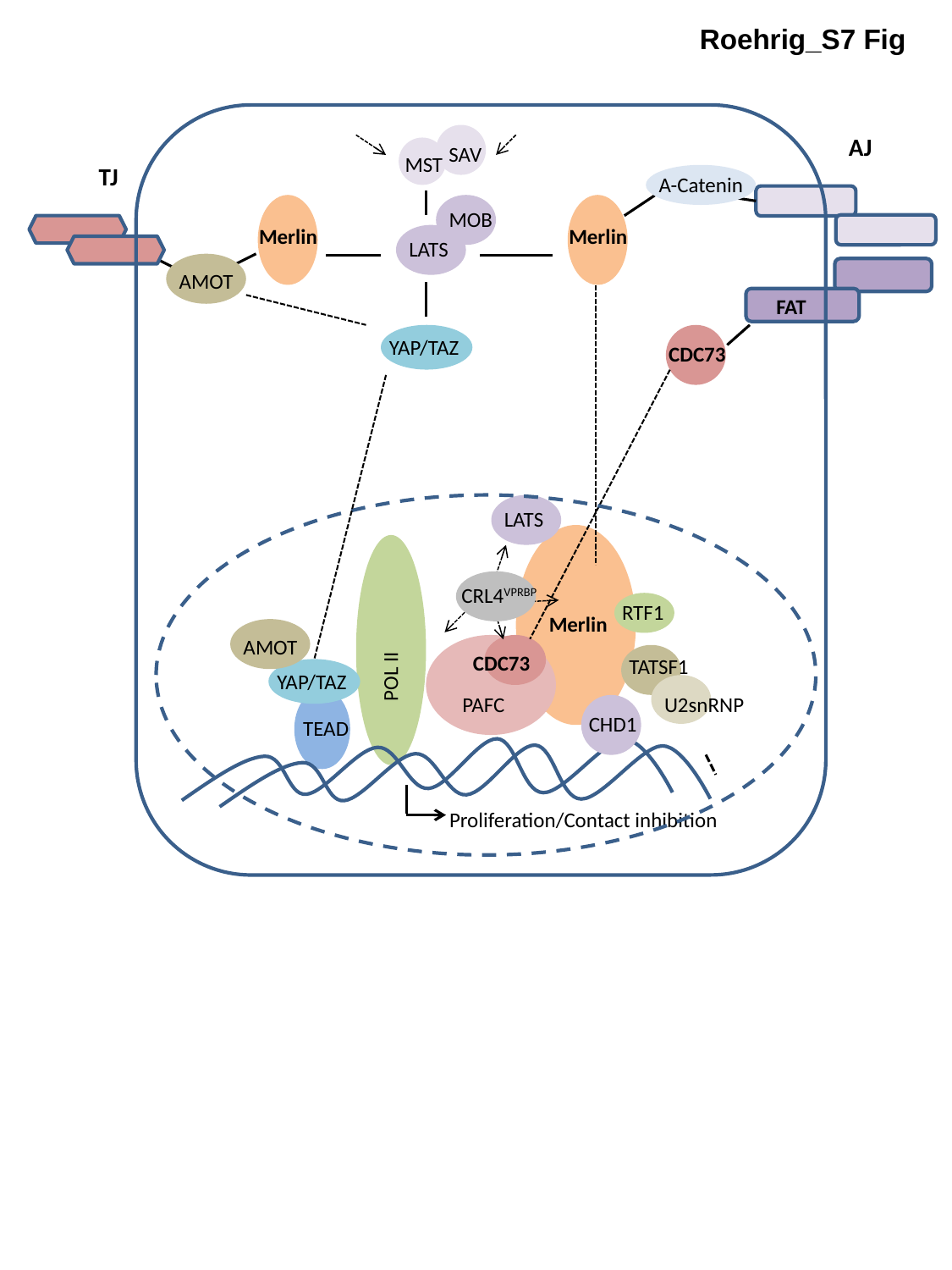

Roehrig_S7 Fig
AJ
SAV
MST
TJ
A-Catenin
Merlin
MOB
Merlin
LATS
AMOT
FAT
YAP/TAZ
CDC73
LATS
CRL4VPRBP
RTF1
Merlin
AMOT
PAFC
CDC73
TATSF1
U2snRNP
POL II
YAP/TAZ
CHD1
TEAD
Proliferation/Contact inhibition
